# The volume of ^99m^Tc sulfur colloid SPET‐defined active bone marrow can predict grade 3 or higher acute hematologic toxicity in locally advanced cervical cancer patients who receive chemoradiotherapy

**DOI:** 10.1002/cam4.2601

**Published:** 2019-10-16

**Authors:** Shan‐Bing Wang, Jia‐Pei Liu, Kai‐Jian Lei, Yu‐Ming Jia, Yan Xu, Jin‐Feng Rong, Chun‐Xiu Wang

**Affiliations:** ^1^ Department of Oncology The Second People's Hospital of Yibin City Yibin Sichuan China; ^2^ Department of Laboratory Medicine The Second People's Hospital of Yibin City Yibin Sichuan China

**Keywords:** active bone marrow, cervical cancer, hematologic toxicity, SPET

## Abstract

**Background:**

The purpose of the current study was to evaluate whether radiation dose‐volume metrics to technetium‐99m (^99m^Tc) sulfur colloid single‐photon emission tomography (SPET)‐defined active bone marrow (ABM) subregions can more accurately predict acute hematologic toxicity in locally advanced cervical cancer patients who receive chemoradiotherapy than conventional dosimetric parameters.

**Methods and materials:**

Thirty‐nine patients with stage IB2‐III cervical cancer who underwent ^99m^Tc sulfur colloid SPET imaging before treatment with cisplatin‐based chemoradiation between January 2017 and March 2018 were analyzed. The total bone marrow (TBM) volume was defined as the external contours of all bones within the vertebral bodies from L4 to the coccyx, the pelvic bones, and the proximal femoral heads. The ABM volume was defined by SPET as the subregion of TBM with a nuclide uptake value greater than or equal to the mean total body nuclide uptake value. Student's *t* test was used to test for statistical significance between TBM and ABM dose‐volume metrics. Receiver operating characteristic (ROC) curves were generated to compare the predictors of grade 3 or higher (grade 3+) hematologic toxicity.

**Results:**

The mean ABM‐V40 (23.22% ± 7.65%) and ABM‐V30 (45.28% ± 9.20%) were significantly lower than the mean TBM‐V40 (33.06% ± 6.72%) and TBM‐V30 (53.08% ± 7.77%), respectively (*t* = 5.78, *P* = .001) (*t* = 4.13, *P* = .001). The ABM volume (<387.5 cm^3^, AUC = 0.928, *P* = .001), ABM‐V30 (>46.5%, AUC = 0.875, *P* = .001), and ABM‐V40 (>23.5%, AUC = 0.858, *P* = .001) can predict the occurrence of grade 3+ hematologic toxicity. Among patients with an ABM volume < 387.5 cm^3^, 16/19 (84.2%) had grade 3+ hematologic toxicity compared to 3/20 (15%) with an ABM volume > 387.5 cm^3^.

**Conclusions:**

The ABM volume (<387.5 cm^3^) may be a better predictor of hematologic toxicity than conventional dose‐volume metrics, but this finding needs to be further evaluated.

## Graphical abstract

The mean ABM‐V40 and ABM‐V30 were decreased by 9.84% and 7.8%, respectively, compared with mean TBM‐V40 and TBM‐V30. ABM volume (AUC = 0.928), ABM‐V30 (AUC = 0.875), and ABM‐V40 (AUC = 0.858) can predict the occurrence of grade 3 or higher (grade 3+) hematologic toxicity. ABM volume (<387.5 cm^3^) may be a best predictor of hematologic toxicity than conventional dose‐volume metrics.

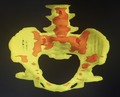

## 1 INTRODUCTION

Locally advanced cervical cancer is a leading cause of gynecological cancer‐associated death in China^1^ and is the fourth most frequent cancer in women worldwide.^2^ Concurrent chemoradiotherapy (CRT) is considered the standard treatment for bulky (Federation of Gynecology and Obstetrics (FIGO) stages IB2 and IIA2) or locally advanced (FIGO stages IIB, III, and IVA) cervical cancer.^3^ Survival benefits from cisplatin‐based CRT in bulky or locally advanced cervical cancer have been observed.^3^, ^4^ However, CRT increases hematologic toxicity,^5^ and 60%‐90% of patients experience grade 2^+^ hematologic toxicity.^6^, ^7^ It reduces the patient's ability to tolerate CRT and the intensity of chemotherapy delivery,^8^ potentially compromising patient survival.^9^ Therefore, reducing hematologic toxicity may improve patient prognosis.

More than 50% of the body's hematopoietically active bone marrow (ABM) is located in the lumbar sacrum, ilium, ischium, pubis, and proximal femur,^10^ which are exposed to pelvic external beam radiation therapy (EBRT) during the treatment of cervical cancer. Radiation causes bone marrow hematopoietic stem cell injury and stromal damage, which is the leading cause of acute hematologic toxicity.^11^

Evidence indicates that bone marrow‐sparing intensity‐modulated radiation therapy (IMRT) can reduce the volume of bone marrow irradiated at high doses compared with traditional radiation therapy.^12^, ^13^ However, the clinical use of such a strategy has been limited, since computed tomography (CT)‐based IMRT cannot identify ABM subregions, and the large avoidance volume compromises planning goals.^10^ ^18^F‐fluorodeoxyglucose positron emission tomography (^18^F‐FDG‐PET) 14 and single‐photon emission CT (SPECT) 15 have been used previously to identify ABM subregions. Previous studies have suggested that technetium‐99m (^99m^Tc) sulfur colloid SPECT defines ABM subregions that can reduce the dose to these areas.^15^ In our study, we hypothesized that the dose to SPECT‐defined ABM is a better predictor of acute hematologic toxicity in locally advanced cervical cancer patients who receive chemoradiotherapy than the dose to total bone marrow (TBM).

## 2 METHODS

### 2.1 Eligibility criteria

We enrolled 39 locally advanced cervical cancer patients in a prospective clinical trial approved by the Ethics Committee of The Second People's Hospital of Yibin (IRB: 2016‐059‐01) and conducted in accordance with the guidelines of the Declaration of Helsinki (Revised 2000). This study is ongoing and is registered with the Chinese Clinical Trial Registry, (http://www.chictr.org.cn), identification number ChiCTR‐IOR‐16010214. All patients signed informed consent in Chinese. Inclusion criteria were as follows: aged 18 to 70 years old, pathologically confirmed locally advanced cervical cancer (FIGO 2008 stages IB2, IIA2, IIB, III, and IVA), Eastern Cooperative Oncology Group (ECOG) performance status 0 or 1, life expectancy >12 months, normal organ and adequate bone marrow function (white blood cell (WBC) count ≥4 × 10^9^/L, absolute neutrophil count (ANC) ≥2 × 10^9^/L, hemoglobin (HGB) ≥110 g/L, platelets (PLT) ≥100 × 10^9^/L, bilirubin ≤2× the upper limit of normal (ULN), aspartate transaminase and alanine transaminase ≤2.5× ULN, and creatinine ≤1.5× ULN). Key exclusion criteria were as follows: a previous history of chemotherapy or pelvic radiotherapy and any cause of a peripheral blood cell abnormality. Forty‐six locally advanced cervical cancer patients were screened for participation; 39 agreed to participate, two refused, two were excluded due to severe anemia, and three withdrew informed consent.

### 2.2 Chemotherapy delivery

Chemotherapy consisting of weekly cisplatin (30‐40 mg/m^2^) alone was given during the time of EBRT. Chemotherapy was suspended under the following conditions: WBC <2×10^9^/L, ANC <1×10^9^/L, HGB <60 g/L, and PLT <50×10^9^/L. If the total WBC count was less than 2 × 10^9^/L, granulocyte colony‐stimulating factor was administered at a dose of 5 mg/kg/d and continued until WBC counts were greater than 4 × 10^9^/L. If the HGB was less than 60 g/L, patients will receive red blood cell transfusion therapy.

All patients planned to receive five cycles of cisplatin; six (15.4%) patients received four cycles, and 33 (84.6%) patients received five cycles of cisplatin.

### 2.3 SPECT scanning and radiation simulation

**Figure 1:**
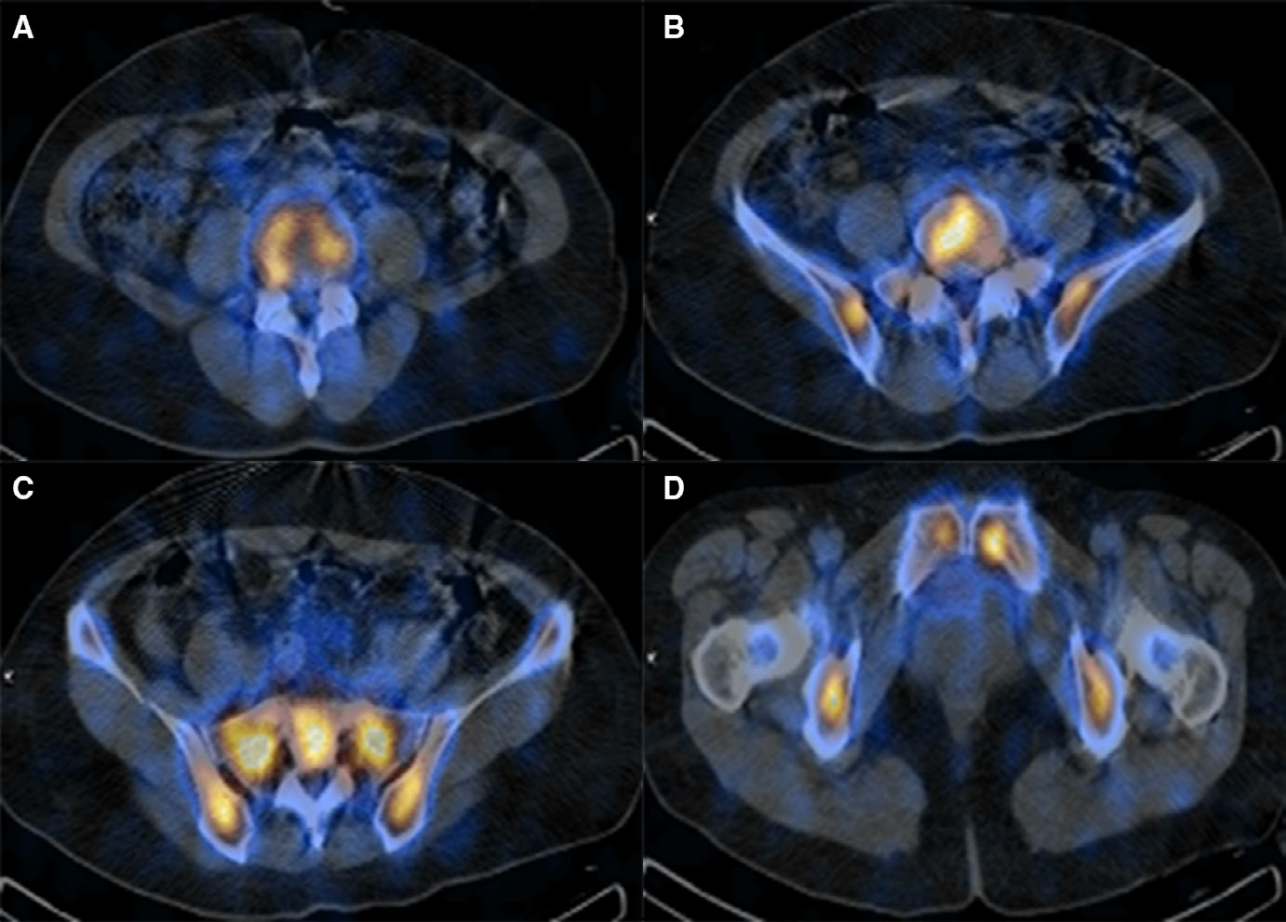
The representative image of the SPECT images fused into CT images. A, L4 vertebral bodies; B, L5 vertebral bodies and iliac crests; C, sacrum and iliac crests; D, pubic symphysis

All patients underwent SPECT/CT scan 1 week before CRT. Thirty minutes before scanning, each patient was given an intravenous injection of Tc‐99m sulfur colloid (0.2 mci/kg). Patients underwent SPECT/CT (GE Infinia Hawkeye 4) imaging in a supine position. Bone marrow imaging from the L3‐4 interspace to the ischial tuberosities was performed using a 0.5‐cm slice thickness. All patients underwent standard CT simulation (TOSHIBA 16‐slice Activion) in the supine position with custom immobilization using a 0.5‐cm slice thickness. The SPECT images were fused into CT simulation images with the Elekta Focal contouring system (Elekta AB). To ensure the accuracy of image fusion, all patients underwent standard CT simulation and SPECT/CT scan with the immobilization customized cradles. Automatic rigid registration based on the bone structure of the two sets of images, manually check the bone structure coincidence degree of the two groups of images, and the deviation can be manually moved or rotated for registration. Figure 1 shows representative image of the fused SPECT‐CT images.

### 2.4 Target delineation and radiation planning and delivery

The clinical target volume (CTV) covers the uterus, cervix, gross tumor parametria, adequate vaginal margin from the gross disease (at least 3 cm), and regional nodes (common, internal, and external iliac nodes, obturator nodes, bilateral groin nodes; in patients with lower 1/3 vaginal involvement). The CTV was extended uniformly by 6 mm in every direction to generate the planning target volume (PTV). The small bowel, rectum, bladder, femoral heads, and bone marrow were delineated as organs at risk. Volumetric‐modulated arc therapy (VMAT) was produced by the Monaco treatment planning system (Elekta AB, Stockholm, Sweden). The PTV received 45 Gy in 25 fractions. Intracavitary, high‐dose rate (HDR) Cobalt‐60 brachytherapy was delivered twice weekly after receiving EBRT. The typical point A prescribed dose was five fractions of 6 Gy per fraction at a HDR. Patients with pelvic lymph nodes received boosts of an additional 10‐20 Gy, lymph nodes boosts was administrated 2 weeks after the end of CRT. The key organ‐at risk dosimetric constraints were the rectum and bladder volume receiving 45 Gy (V_45_) <45% and the bowel volume receiving <10%, respectively. All VMAT plans were normalized to cover approximately 98% of the PTV with 45Gy.

### 2.5 ABM and TBM delineation

The external contours of all bones within the vertebral bodies from L4 to the coccyx, the pelvic bones, and the proximal femoral heads were delineated on the planning CT under same setting (window width, 1000HU and window level,‐350HU) to defined as the TBM volume). The subregion in the TBM with a nuclide uptake value greater than or equal to the mean total body nuclide uptake value was delineated on the planning CT to define the ABM volume.

### 2.6 Hematologic toxicity

All subjects treated with CRT had complete blood counts weekly. The WBC, ANC, HGB, and PLT counts within 2 weeks after the completion of CRT were the primary study endpoints. Hematologic toxicity was assessed with the Radiation Therapy Oncology Group acute radiation morbidity scoring criteria (CTCAE 3.0).^16^ Grade 3 or higher (grade 3+) hematologic toxicity was defined as any grade 3+ toxicity for HGB, ANC, PLT, and WBC.

### 2.7 Statistical analysis

For every patient, the volume (cm^3^), mean dose, and volume receiving 10 Gy (V10), volume receiving 20 Gy (V20), volume receiving 30 Gy (V30), and volume receiving 40 Gy (V40) for ABM and TBM were compared by the *t* test. Receiver operating characteristic (ROC) curves were generated to compare the predictors of grade 3+ hematologic toxicity. All statistical analyses were performed using SPSS version 22.0 (IBM, Armonk, NY), and *P* values ≤ .05 were considered statistically significant.

## 3 RESULTS

The patient demographics and tumor characteristics are summarized in Table 1. The mean age of the patients was 51.4 ± 9.0 years. Six patients were FIGO stage IB2, 10 patients were FIGO stage IIA2, 15 patients were FIGO stage IIB, and eight patients were FIGO stage III. Of the 39 patients, 37 had squamous cell carcinoma and two had adenocarcinoma.

**Table 1:** Patient and tumor characteristics

Characteristic		Value
Patients	39	
Median age, y (rang)	51 (32‐70)	
Median dose(Gy)	45 (45‐60)	
Mean BMI (kg/m^2^)	23.2 ± 5.2	
FIGO stage		
IB2	6 (15.4%）	
IIA2	10 (25.6%)	
IIB	15 (38.5%)	
IIIA	5 (12.8%)	
IIIB	3 (7.7%)	
Cycles of cisplatin		
≤4	6 (15.4%)	
＞4	33 (84.6%)	
Histology		
Squamous cell carcinoma	37 (94.9%)	
Adenocarcinoma	2 (5.1%)	
Rate of grade 3 or higher hemotoxicity	19 (48.7%)	
Leukopenia	18 (46.2%)	
Neutropenia	1 (2.5%)	
Anemia	0 (0%)	
Thrombocytopenia	0 (0%)	

Abbreviations: BMI, body mass index; FIGO, Federation of Gynecology and Obstetrics; SD, standard deviation

**Figure 2:**
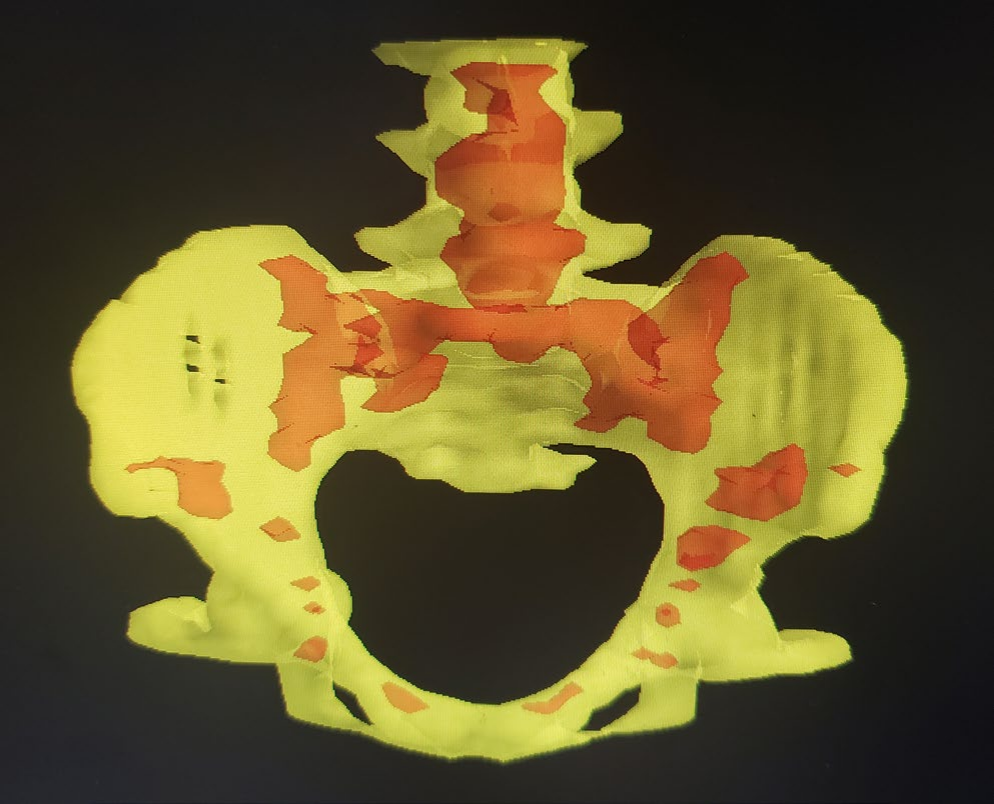
Red represents the distribution of active bone marrow, yellow represents the total bone marrow

The ABM volumes, TBM volumes, and radiation dose‐volume metrics are shown in Table 2. The mean TBM volume (953.59 ± 155.54 cm^3^) was significantly higher than the mean ABM volume (354.60 ± 172.89 cm^3^) (*t* = −17.19, *P* = .001). ABM was most commonly situated in the lumbar vertebrae, sacrum, and pubic bones (Figure 2). The mean ABM‐V40 (23.22% ± 7.65%) and ABM‐V30 (45.28% ± 9.20%) were significantly lower than the mean TBM‐V40 (33.06% ± 6.72%) and TBM‐V30 (53.08% ± 7.77%) (*t* = 5.78, *P* = .001) (t = 4.13, *P* = .001), but there was no significant difference between ABM and TBM in the dose‐volume of low‐dose radiation (V10 and V20) or mean dose.

**Table 2:** Active vs total bone marrow volume and dosimetric parameters

	TBM	ABM	*t* value	*P* value
Volume (cm^3^)	953.59 ± 155.54	354.60±172.89	−17.19	.001
Mean dose (Gy)	28.90 ± 9.03	30.36 ± 6.51	−1.70	.097
V10%	90.10 ± 4.65	90.21 ± 4.49	−0.13	.900
V20%	81.41 ± 5.49	79.59 ± 5.07	1.59	.118
V30%	53.08 ± 7.77	45.28 ± 9.20	4.13	.001
V40%	33.06 ± 6.72	23.22 ± 7.65	5.78	.001

Abbreviations: ABM, active vs total bone marrow; TBM, total bone marrow.

Of the 39 patients, 19 (48.7%) suffered from acute grade 3‐4 hematologic toxicity. Among the patients with acute grades 3‐4 hematologic toxicity, 18 suffered from acute grades 3‐4 leukopenia, and one patient suffered from acute grades 3‐4 neutropenia.

**Figure 3:**
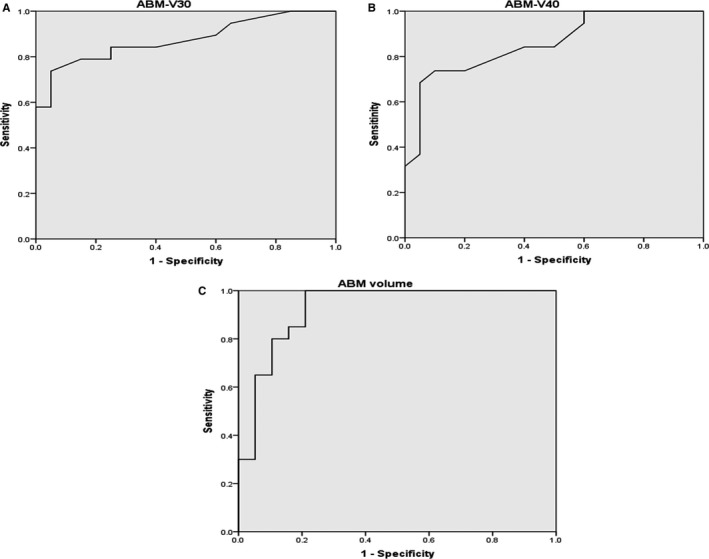
The receiver operating characteristic (ROC) curves of ABM‐V30 (A), ABM‐V40 (B), and ABM volume (C)

Table 3 summarizes the results of the ROC curve analysis. The mean dose, ABM‐V10, ABM‐V20 TBM‐V10, TBM‐V20, TBM‐V30, TBM‐V40, and mean TBM volumes cannot predict grade 3+ hematologic toxicity. The ABM volume (<387.5 cm^3^, AUC = 0.928, *P* = .001), ABM‐V30 (>46.5%, AUC = 0.875, *P* = .001), and ABM‐V40 (>23.5%, AUC = 0.858, *P* = .001) can predict the occurrence of 3+ hematologic toxicity (Figure 3), with sensitivities of 84.2%, 73.7%, and 72.7%, respectively, and with specificities of 85%, 95%, and 90%, respectively. In patients with an ABM volume <387.5 cm^3^, 16/19 (84.2%) had grade 3+ hematologic toxicity compared to 3/20 (15%) with an ABM volume >387.5 cm^3^.

**Table 3:** Receiver operating characteristic curves analysis for grade 3 or higher (grade 3+) hematologic toxicity

Parameter	Cut off value	AUC	*P* value	Sensitivity	Specificity
TBM V10		0.433	.474		
TBM V20		0.422	.407		
TBM V30		0.500	1.000		
TBM V40		0.529	.757		
ABM V10		0.572	.440		
ABM V20		0.438	.509		
ABM V30	>46.5%	0.875	.001	73.7%	95%
ABM V40	>23.5%	0.858	.001	72.7%	90%
TBM mean dose		0.666	.077		
ABM mean dose		0.593	.319		
TBM volume		0.512	.895		
ABM volume	<387.5 cm^3^	0.928	.001	84.2%	85%

## 4 DISCUSSION

In the current study, we observed that the ABM volume, ABM‐V30, and ABM‐V40 were significantly correlated with the development of grade 3+ hematologic toxicity. By contrast, there was no significant association between the radiation dosimetric parameters of TBM and the development of grade 3+ hematologic toxicity. Furthermore, we found that the ABM volume was the best predictor of grade 3+ hematologic toxicity. To the best of our knowledge, the current study is the first to assess the radiation dosimetric parameters of ^99m^Tc sulfur colloid single‐photon emission tomography (SPET) to define ABM in the prediction of grade 3+ acute hematologic toxicity in locally advanced cervical cancer patients who receive chemoradiotherapy.

Hematologic toxicity is the most common side effect in locally advanced cervical cancer patients treated with CRT.^17^ Previous studies have shown that the incidence of grade 3+ acute hematologic toxicity was 60%‐77%.^7^, ^11^, ^18^ In our study, 19 (48.7%) patients suffered from grade 3+ acute hematologic toxicity. Hematologic toxicity is the main cause of delayed or missed chemotherapy cycles and treatment interruption, which is correlated with poor disease control and survival.^8^, ^9^, ^18^ Some studies have demonstrated that treatment intensification may improve survival in locally advanced cervical cancer patients.^17^, ^19^ However, the incidence of grade 3+ acute hematologic toxicity was 72%, which led to treatment break.^19^ Reducing the incidence of acute hematologic toxicity may increase the intensity of treatment and improve survival.

Prior research has shown that TBM radiation dosimetric parameters predicted the occurrence of acute hematologic toxicity in locally advanced cervical cancer patients who received chemoradiotherapy,^20^, ^21^, ^22^ but the results are not consistent. Some studies proposed that the volume of TBM receiving 10 and 20 Gy can exactly predict the development of acute hematologic toxicity.^20^, ^21^ In contrast, Klopp et al found that the volume of TBM receiving 40 Gy, rather than the volume of TBM receiving 10 and 20 Gy, exactly predicted the development of acute hematologic toxicity.^22^ However, CT images cannot distinguish between active and inactive bone marrow regions, which leads to a large volume of bone marrow, potentially compromising target coverage and affecting other organs at risk.^23^ The accurate identification of functionally ABM within the pelvic bones reduces the avoidance volumes and could help bone marrow sparing and improve the predictive value of acute hematologic toxicity.

Currently, various imaging modalities, such as ^18^F‐FDG‐PET,^14^ magnetic resonance imaging (MRI),^24^ and ^99m^Tc sulfur colloid SPET,^15^ are used to distinguish functionally ABM. However, there is a lack of a consensus on optimal imaging modalities for the accurate identification of functionally ABM. Studies by Roeske et al revealed that ^99m^Tc sulfur colloid SPET could accurately identify ABM, and ABM‐sparing IMRT planning reduced the volume of ABM irradiated at high doses compared to TBM‐sparing IMRT planning.^15^ However, there was no reported correlation between dosimetric parameters and ^99m^Tc sulfur colloid SPET‐defined ABM and acute hematologic toxicity in locally advanced cervical cancer patients who received chemoradiotherapy. In our study, we found that the mean ABM‐V40 and ABM‐V30 were decreased by 9.84% and 7.8%, respectively, compared with the mean TBM‐V40 and TBM‐V30. In addition, our research also shows that the ABM volume, ABM‐V30, and ABM‐V40 were significantly correlated with the development of grade 3+ hematologic toxicity. More interestingly, we observed that the ABM volume could better predict the occurrence of grade 3+ acute hematological toxicity compared to ABM‐V30 and ABM‐V40. Patients in this study with an ABM volume >387.5 cm^3^ at baseline seem to be at the lowest risk for the occurrence of acute hematologic toxicity. Similarly, Zhou et al and Khullar et al also showed that the volume of ^18^F‐FDG‐PET‐defined ABM may be a best predictor of acute hematological toxicity than conventional dosimetric parameters.^7^, ^11^ Therefore, the critical volume may be important in predicting the occurrence of acute hematologic toxicity. In 2005, based on the liver as a parallel organ, Schefter et al applied a normal liver critical volume to predict radiation‐induced liver disease. This research demonstrated that a normal liver volume ≥700 mL receiving a low dose correlated with a low rate of radiation‐induced liver disease after stereotactic body radiation therapy.^25^ Similar to the liver, the bone marrow is considered a functionally parallel organ^7^; as long as there are enough active functional cells, hematological toxicity will not occur. Therefore, we have adequate reason to believe that the ABM volume is important in predicting acute hematological toxicity, but this model needs to be further evaluated.

Despite, our study find that the ABM volume, ABM‐V30, and ABM‐V40 were significantly correlated with the development of grade 3+ hematologic toxicity. But, this study has some limitations. First, the data in our study were derived from a single center study, and further multi‐center clinical trials need to be carried out to confirm our findings. Moreover, severe anemia, more than 70 years old, and worse performance status patients were excluded from this study, so it is uncertain whether our findings are applicable to these patients.

In conclusion, our study supports the hypothesis that the ABM volume, ABM‐V30, and ABM‐V40 are significantly correlated with the development of grade 3+ hematologic toxicity. Techniques to limit ABM irradiation may reduce acute hematological toxicity in locally advanced cervical cancer patients. In our ongoing study (ChiCTR‐IOR‐16010214), we are testing whether ^99m^Tc sulfur colloid SPET‐defined ABM‐sparing VMAT can reduce the risk of acute hematological toxicity for patients with locally advanced cervical cancer.

## CONFLICT OF INTEREST

The authors of this manuscript declare no conflict of interest.
